# Vitamin B12 injection plus traditional Chinese medicine techniques for meralgia paresthetica: a randomized controlled trial

**DOI:** 10.3389/fmed.2025.1670225

**Published:** 2026-01-12

**Authors:** Hongxia Guan, Yong Shi, Xia Zhao

**Affiliations:** 1 Department of Traditional Chinese Medicine, Changchun University Hospital, Changchun, Jilin, China; 2 School of Special Education, Changchun University, Changchun, Jilin, China; 3 Department of Acupuncture and Moxibustion, Dalian Jinzhou District Hospital of Traditional Chinese Medicine, Dalian, Liaoning, China

**Keywords:** cupping, cutaneous needle, lateral femoral cutaneous neuritis, numbness, pain, pulling, vitamin B12

## Abstract

**Background:**

Traditional Chinese medicine (TCM) comprehensive therapy has become the mainstream treatment method for lateral femoral cutaneous neuritis. Vitamin B12 plays an important role in neuropathic diseases. This study aimed to explore the clinical effect of vitamin B12 injection combined with TCM technology in the treatment of lateral femoral cutaneous neuritis.

**Methods:**

From January 2022 to January 2024, 126 patients with lateral femoral cutaneous neuritis were selected from our hospital and then randomly divided into a study group and control group. The control group received the methods of pulling, paravertebral positive spot cutaneous needle tapping, and cupping. The study group received vitamin B12 injections on the basis of the methods of pulling, paravertebral positive spot cutaneous needle tapping, and cupping.

**Results:**

After therapy, the pain score and the numbness score in the study group were lower relative to the control group. The total effective rate in the study group was better relative to the control group. The recurrence rate and the incidence of adverse reactions in the study group were lower relative to the control group.

**Conclusion:**

Vitamin B12 injection combined with pulling, paravertebral positive spot cutaneous needle tapping, and cupping has an effective clinical effect in the treatment of lateral femoral cutaneous neuritis.

## Introduction

Lateral femoral cutaneous neuritis is also known as meralgia paresthetica or Roth syndrome. An American study published in 2011 reported that the incidence of lateral femoral cutaneous neuritis is approximately 3.6 per 10,000 person-years (1). Lateral femoral cutaneous neuritis belongs to the category of “muscle arthralgia” and “skin arthralgia” of traditional Chinese medicine (TCM), primarily due to Yang deficiency, invasion of wind-cold dampness evil, blocking meridians, and Qi and blood stasis (2).

From the perspective of modern medicine, the lateral femoral cutaneous nerve originates from the posterior femoris of the anterior branch of the second and third lumbar nerves. It descends obliquely from the lateral margin of the psoas major, traversing the iliac muscle to the anterior iliac superior spine or to its medial side, penetrating the sartorius muscle to pass through the fascia lata and the superficial fascia, and distributing in the anterolateral skin of the thigh (1). This condition is most commonly caused by the compression of the lateral femoral cutaneous nerve in the medial superior iliac spine through the bone-fiber canal at the inguinal ligament or through the fascia lata (3). It is common in middle-aged and elderly obese men, pregnant women, and those who wear tight clothes (4). Lateral femoral cutaneous neuritis is characterized by abnormal skin sensations on the anterolateral 2/3 of the thigh, such as pain, numbness, burning sensation, and formant sensation, and can occur in 7–35% of patients with leg discomfort (5).

For the treatment of this disease, Western medicine primarily uses glucocorticoids and other nutritional nerve, eliminates inflammation, or uses analgesics, local closure temporary analgesia, and other methods, but the effect is not good and there is no specific drug treatment (6).

In contrast, TCM offers several advantages in the treatment of lateral femoral cutaneous neuritis, including quick action, safety, simplicity, small adverse reactions, and low cost (7). Comprehensive TCM therapy has emerged as the mainstream approach for treating this condition. Extensive clinical practice has demonstrated that it can accurately relieve patients’ symptoms such as pain and paresthesia (8).

TCM techniques, such as pulling, paravertebral positive spot cutaneous needle tapping, and cupping, have been increasingly investigated for their potential in managing neuropathic pain. The “pulling method” in TCM involves using the thumb and index fingers, or adding the middle finger, to quickly and repeatedly pinch the affected skin vigorously, without the need for other tools. This method aims to relieve nerve compression through manual traction (9). Cutaneous needles in the appropriate technology of TCM are multi-needle shallow needling devices used for tapping acupoints and other parts of the skin, acting on the epidermis, without damaging capillaries (10).

Cupping therapy, a cornerstone of TCM with a history spanning over 2,000 years, is a crucial treatment in our research (11). It creates negative pressure via suction cups applied to the skin. This negative pressure is believed to improve local microcirculation, which helps to increase the delivery of oxygen and nutrients to the affected tissues while removing metabolic waste products (12, 13). It also alleviates muscle tension, thereby reducing the pressure on nerves and blood vessels in the area (14). Moreover, cupping therapy may modulate pain pathways through the release of endogenous opioids and anti-inflammatory cytokines, thereby providing pain relief (15). Recent meta-analyses have demonstrated that cupping therapy effectively reduces pain intensity in conditions such as low back pain and peripheral neuropathies, with efficacy comparable to or complementary with pharmacological interventions and a low risk of adverse events (16, 17). The treatment is easy to operate and is well-known to the public, with relatively little pain during the process, which helps to avoid patients’ fear and makes it easily accepted by patients.

Vitamin B12, also known as cobalamin, is one of the basic substances required to maintain the integrity of the human nerve myelin sheath (18). It has a definite effect in maintaining nerve myelin function and metabolism (19). It promotes the conversion of methylmalonic acid to succinic acid and participates in the tricarboxylic acid cycle, effectively playing the role of neuronutrition, accelerating the synthesis of nerve myelin lipids, and promoting the good function of myelin nerve fibers (20). Accumulating evidence has proven that vitamin B12 plays an important role in the treatment of neuropathic diseases (21).

While vitamin B12 addresses neuropathy by providing essential nutrients for nerve function and repair, TCM techniques such as cupping reduce inflammation and improve local tissue conditions. To the best of our knowledge, no randomized controlled trials (RCTs) have combined vitamin B12 injection with pulling, paravertebral positive spot cutaneous needle tapping, and cupping for the treatment of meralgia paresthetica. This gap in research is precisely what our trial aims to address.

In our study, we aimed to explore the clinical effect of vitamin B12 injection combined with pulling, paravertebral positive spot cutaneous needle tapping, and cupping in the treatment of lateral femoral cutaneous neuritis.

## Methods

### General data

**Figure 1:**
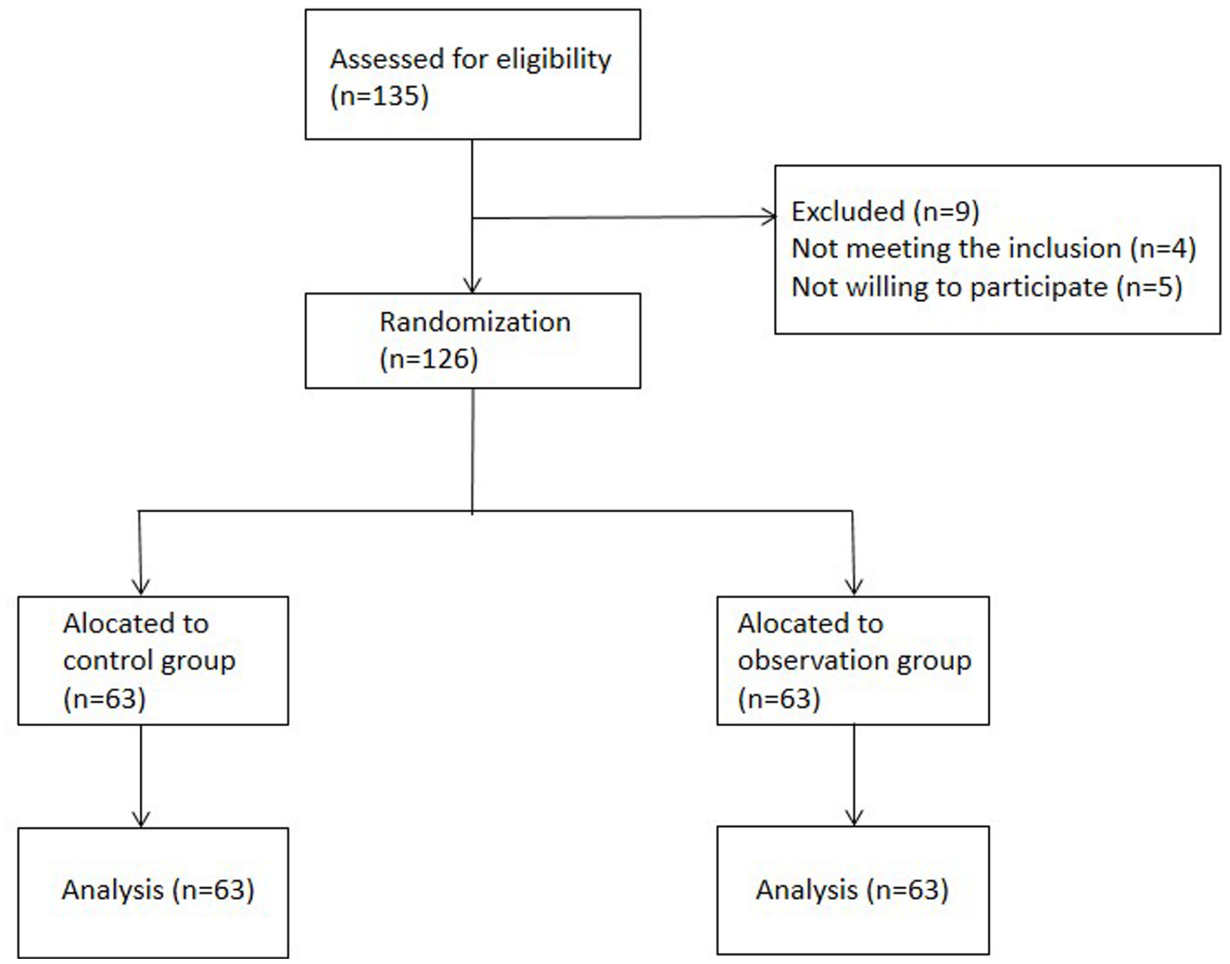
CONSORT flowchart.

From January 2022 to January 2024, 126 patients with lateral femoral cutaneous neuritis were selected from our hospital. The CONSORT flowchart is shown in Figure 1.

Diagnostic criteria: The patient had obvious numbness, tingling, and burning sensations in the lower 2/3 of the anterolateral thigh. The majority of the thigh were parochial, and the severe ones are persistent, unable to stand or walk for a long time, and would be significantly reduced after a short rest. After examination, it was found that there were tender points on the medial or lower side of the anterior superior iliac ridge. There were often areas of different sizes and shapes on the anterolateral thigh skin. In addition, the patient did not have obvious muscle atrophy or movement disorders, and there was an obvious tendon reflex.

The inclusion criteria were as follows: (1) patients that met the diagnostic criteria of lateral femoral cutaneous neuritis; (2) those aged between 18 and 70 years; (3) those with unilateral lateral femoral cutaneous neuritis; (4) those that stopped taking medication and other treatments within 1 week before enrollment.

The exclusion criteria were as follows: (1) patients with wounds, ulcers, and infections on the skin of the lesion site of the lateral thigh; (2) those with high lumbar disc herniation; (3) neuropathies secondary to spinal fractures and dislocations, tumors, pelvic and hip bone lesions, or diabetes; (4) pregnant and lactating women; (5) those with cardiovascular and cerebrovascular diseases, liver and kidney function injury, and mental illness; and (6) those who were prone to co-infection and allergy. All patients were informed of this study and signed informed consent.

### Randomization and blinding

Patients were randomized using a random number table method. Specifically, we first generated a series of random numbers using a standard random number table. Each patient was then assigned a unique sequential number upon enrollment. Subsequently, these sequential numbers were matched with the randomly generated numbers from the table. Based on whether the matched random number was odd or even, patients were allocated to either the study group or the control group, ensuring an equal probability of assignment to each group. As a result, 63 patients were assigned to the study group and 63 patients to the control group.

In terms of blinding, due to the nature of the interventions and the study design, it was not feasible to blind the patients to their group assignments. However, to minimize potential bias, the outcome assessors were blinded to the group’s allocations. The outcome assessors were independent researchers who were not involved in patient recruitment, randomization, or intervention processes. They evaluated the patients’ outcomes based on predefined criteria without any knowledge of which group the patients belonged to.

### Treatment methods

Patients in both groups were administered mecobalamine tablets 500 μg/time, twice daily, and ibuprofen capsules 300 mg/time, twice daily for 20 days.

Patients in the control group underwent pulling therapy, paravertebral positive spot cutaneous needle tapping, and cupping.

For the pulling method, the doctor used the thumb and index fingers, or the middle finger, to quickly and forcefully pinch the affected skin of the patient with moderate force until the skin become purplish-red or damp red. The patient reported that the heat penetrated the skin and underlying muscles, producing a sense of relaxation and comfort in the affected area. Treatment was administered once daily for five session of a course of treatment, and if the symptoms gradually declined during the treatment, it could be reduced to once every 2 days.

For the paravertebral positive spot cutaneous needle tapping method, the doctor observed the lumbar spine and sacral paravertebral muscles for abnormalities, found abnormal positive spots with protrusions or depressions, and used a cutaneous needle for tapping. Treatment was administered once daily for five sessions of a course of treatment.

For the cupping method, cupping was performed at the cutaneous needle tapping sites for 10 min per session, once daily, 5 times per course. If symptoms resolved after the first course, a second course was administered to consolidate the effect.

Patients received vitamin B12 injection combined with the methods of pulling, paravertebral positive spot cutaneous needle tapping, and cupping. The methods of pulling, paravertebral positive spot cutaneous needle tapping, and cupping were the same as the control group. For vitamin B12 injection, 500 μg/mL of vitamin B12 (Sinopharm Group Rongsheng Pharmaceutical Co., LTD.) was extracted with a 5-ml disposable syringe for later use. Procedure: The needle was inserted at the marked point 1 cm inside the anterior superior iliac spine of the patient. The needle was introduced at an angle of 40°–60° and advanced slowly along the surface of the iliac bone toward the tail end until a breakthrough sensation of the was felt, namely the iliac fascia. The needle was fixed, and the prepared vitamin B12 was injected after blood was withdrawn. After the injection, the needle was removed, sterile gauze was applied at the injection site until hemostasis was achieved, after which a sterile dressing was applied. Patients were instructed to keep the injection site dry for 24 h. The treatment was performed once a week.

### Observation indicators

1. Visual analogue scale (VAS) was used to evaluate the pain degree of patients. It consists of a horizontal line, 10 cm in length, with endpoints labeled “0” and “10.” The left-hand endpoint (0) represents “no pain at all,” while the right-hand endpoint (10) indicates “the most severe pain imaginable.” Patients were clearly instructed on how to use the VAS. They were asked to mark a point on the line that best represented their current level of pain based on their own subjective feelings. The distance from the “0” end to the marked point was then measured using a ruler and converted into a numerical score ranging from 0 to 10. A higher score on the VAS corresponds to a more severe level of pain.
2. The degree of numbness of patients was evaluated using the VAS score. Similar to the pain assessment, the VAS for numbness is a 10-cm horizontal line. The left-hand end (0) is labeled as “no numbness,” and the right-hand end (10) is defined as “severe numbness that is unbearable.” Patients were provided detailed instructions on how to rate their numbness using this scale. They were asked to mark a point on the line according to their own subjective perception of the numbness they were experiencing. The distance from the “0” mark to the patient-marked point was measured and converted into a score from 0 to 10. A higher score indicates a greater degree of numbness.
3. Clinical efficacy. Cured: The clinical symptoms of the affected area completely disappeared, and the superficial sensation of the affected skin returned to normal. Obvious effect: Symptoms such as pain and numbness in the affected area disappeared or were significantly reduced, but the mild pain and numbness occurred after prolonged sitting or fatigue. The shallow sensation on the skin was significantly restored, and the area of the lesion was significantly reduced. Effective: Pain, numbness, and other symptoms of the affected area were alleviated, and shallow sensation on the skin was restored, and the area of the lesion was reduced. Ineffective: Symptoms and signs remained without significant change. Total effective rate = (cured + obvious effect + effective) cases/total cases ×100%.
4. Patients were followed for 6 months, and the recurrence rate was compared in two groups.
5. The incidence of adverse reactions, including nausea, vomiting, and loss of appetite, in two groups was recorded.

### Statistical analysis

SPSS 24.0 statistical software was adopted for data analysis. Measurement data were expressed as (Mean ± Standard deviation), and the t-test was adopted for comparison. Count data were expressed as (*n*, %), and χ^2^ test was used for comparison. A *p*-value of <0.05 indicated statistical significance.

## Results

### Baseline data in two groups

Compared with the general data of the two groups, there was no statistical significance (*p* > 0.05, Table 1), indicating comparability.

**Table 1:** General data of two groups.

Groups	Sex (male/female)	Age (years)	Course of disease (months)
Control group (*n* = 63)	33/30	51.29 ± 14.83	8.62 ± 2.15
Study group (*n* = 63)	32/31	51.32 ± 14.92	8.67 ± 2.23
χ^2^/t	0.031	0.011	0.128
*p*	0.858	0.991	0.898

### Pain VAS scores in two groups

**Figure 2:**
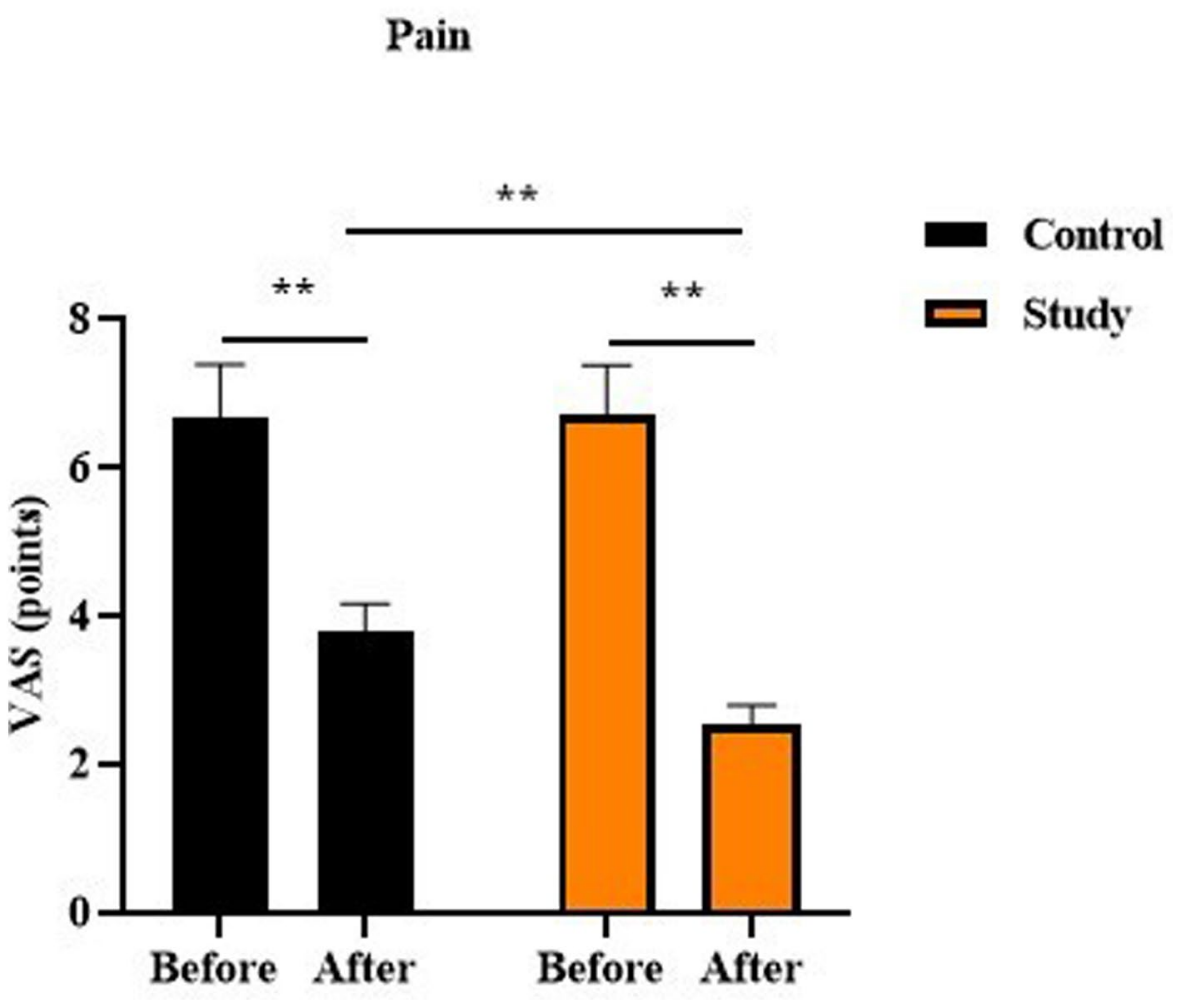
Pain VAS scores in two groups, *p* < 0.01.

Before therapy, no difference was seen in the pain VAS score between two groups (p > 0.05). After therapy, the pain VAS score was reduced in two groups, and the pain VAS score in the study group was lower relative to the control group (*p* < 0.01, Figure 2).

### Numbness VAS score in two groups

**Figure 3:**
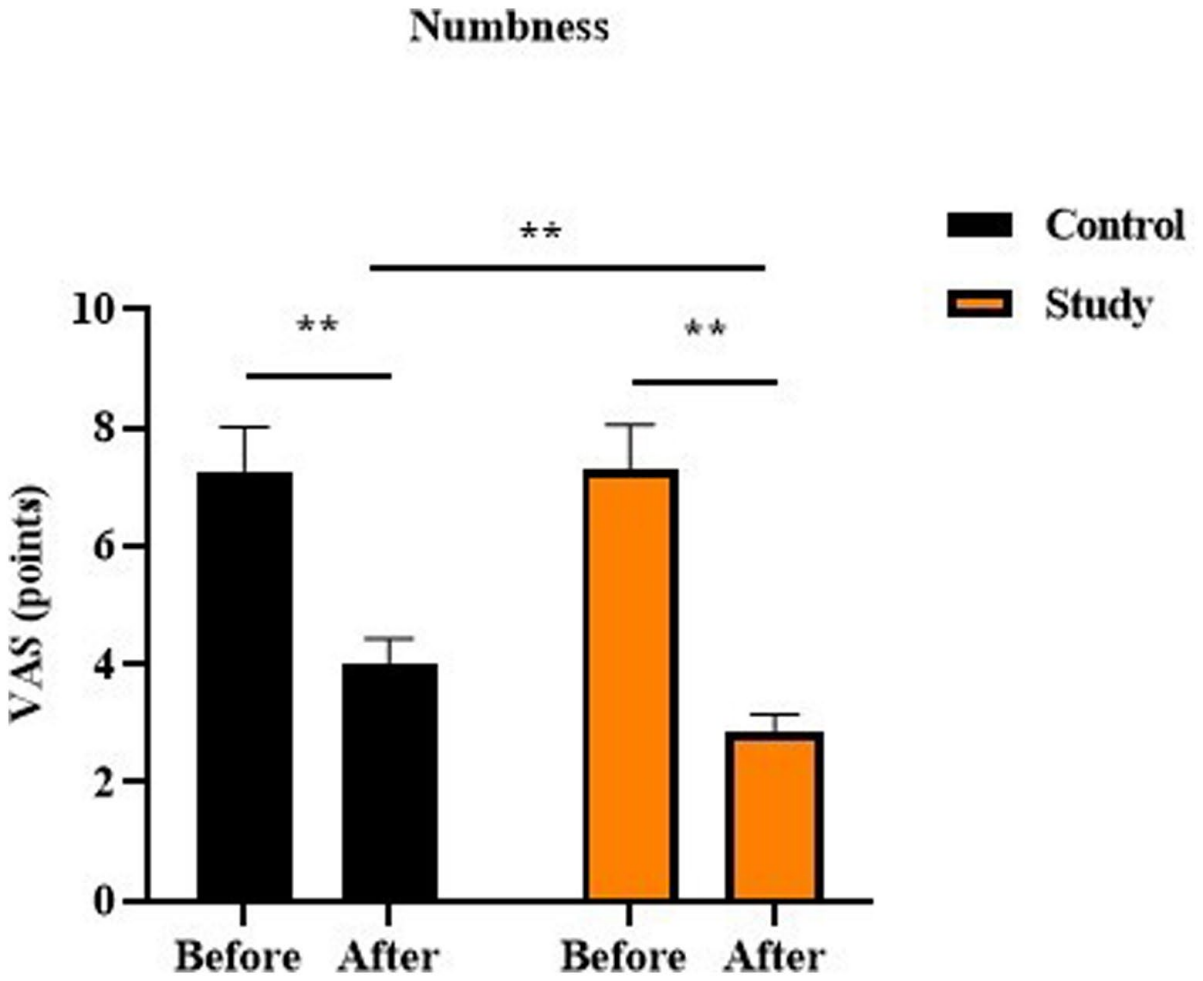
Numbness VAS score in two groups, *p* < 0.01.

Before therapy, no difference was observed in the numbness VAS score between two groups (*p* > 0.05). After therapy, the numbness VAS score was reduced in two groups, and that in the study group was lower relative to the control group (*p* < 0.01, Figure 3).

### Clinical efficacy in two groups

The total effective rate in the study group was better relative to the control group (*p* < 0.05, Table 2).

**Table 2:** Clinical efficacy in two groups.

Groups	Cases	Cured	Obviously effective	Effective	Ineffective	Total effective rate
Control group	63	2	16	33	12	51 (80.95%)
Study group	63	6	33	20	4	59 (93.65%)
*χ*^2^						4.582
*p*						0.032

### Recurrence rate in two groups

The recurrence rate in the study group was lower relative to the control group (p < 0.05, Table 3).

**Table 3:** Recurrence rate in two groups.

Groups	Cases	Number of recurrence	Recurrence rate
Control group	63	8	12.69%
Study group	63	2	3.22%
*χ*^2^		3.910	
*p*		0.048	

### Incidence of adverse reactions in two groups

The incidence of adverse reactions in the study group was lower relative to the control group (*p* < 0.05, Table 4).

**Table 4:** Incidence of adverse reactions.

Groups	Cases	Nausea	Vomiting	Loss of appetite	Total incidence rate
Control group	63	3	2	3	8 (12.69%)
Study group	63	1	0	1	2 (3.22%)
*χ*^2^					3.910
*p*					0.048

## Discussion

In TCM, lateral femoral cutaneous neuritis is classified under the category of “arthralgia” (22). The pathogenesis of the disease can be attributed to the deficiency of positive qi inside and the invasion of wind-cold-dampness evils, which remained in the two percutaneous parts of Foot Yangming and Foot Shaoyang meridians, resulting in the obstruction of the two channels of the muscle, stagnation of Qi and blood for a long time, and numbness (23). The principle of its treatment should primarily be to eliminate blood stasis, to promote regeneration of blood, and to activate channels and collaterals (24).

Clinical drug treatment of lateral femoral cutaneous neuritis has good efficacy, but it is not ideal, and the rate of adverse drug reaction is high, and the recurrence rate is high, so more and more scholars have begun to consider the treatment of TCM (25). The rule of pulling method in TCM involves repeated pinching and pulling of the skin, which can make the skin congested, improve local microcirculation, and play the roles of relaxing the tendons and regulating qi, dispelling wind and cold, clearing heat and dehumidifying, activating blood and removing stasis, and eliminating numbness (26). According to the skin theory, the pulling method acts on the nerve terminal receptors of patients, stimulating the central nervous system to cause excitement, thereby promoting the local blood circulation of patients, alleviating or relieving the state of nerve compression, eliminating the abnormal feeling of the lateral thigh and femoris skin of the affected side, and achieving the purpose of treatment (27). Paravertebral positive spot cutaneous needle tapping is one of the oldest treatment methods in TCM, and its mechanism is to adjust Yin and Yang, dredge channels and collaterals, and harmonize Qi and blood (28). Cupping therapy with heat and negative pressure, applied to the affected area, has the effect of warming the channel to dissipate cold, promoting blood circulation to remove blood stasis, and activating the channels (29).

Vitamin B12, the latest found vitamin, has gained increasing attention due to its essential role in the normal operation of the brain and nervous system and the formation of blood. Vitamin B12 is involved in processes including DNA synthesis and regulation and fatty acid synthesis (30). Vitamin B12 is the largest and most complex vitamin in the body, and its structure is made up of a cyrine ring similar to hemoglobin and chlorophyll (31). In vitamin B12, the active site uses cobalt to bind various chemical groups to form different analogues, including cyanide, hydroxyl, methyl, and 5′-deoxyadenosine. Methyl and 5′-deoxyadenosine are used in the human body to catalyze specific enzyme reactions (32). Vitamin B12 has important functions in nerves, including (1) promoting nerve regeneration: studies have shown that vitamin B12 can help nerve regeneration by inducing axon growth and differentiation of glial cells, thereby improving the difficult nerve healing caused by compression. In addition, vitamin B12 increases brain-derived neurotrophic factor and increases nerve conduction velocity, which also explains part of the nerve regeneration process (33). (2) Analgesic effect: The analgesic mechanism of vitamin B12 is related to a cyclooxygenase involved in inflammation (34). In one study, by artificially creating pain responses in mice, it was observed that vitamin B12 and non-steroidal anti-inflammatory drugs had the same effect of inhibiting cyclooxygenase, thereby concluding the fact that they had a pain-relieving effect (35). Other studies have suggested that vitamin B12 may have both central and peripheral cyclooxygenase-inhibiting properties (36). Vitamin B12 also reduces pain signals by attenuating the action of the capsaicin receptor, a receptor involved in pain processing that responds to heat, acid, and capsaicin (37).

In our study, the results showed that, after treatment, the pain score and the numbness score in the study group were lower than those in the control group, suggesting that vitamin B12 injection combined with pulling, paravertebral positive spot cutaneous needle tapping, and cupping could relieve pain and numbness in the treatment of lateral femoral cutaneous neuritis. Consistently, Furgała et al. suggested that vitamin B12 injections reduced the pain in certain diseases (38). Ahmed et al. discovered that a patient developed numbness and tingling in the distal extremities with subsequent weakness, and early intervention with vitamin B12 supplementation could cause reversal of both central and peripheral nervous system dysfunction (39).

Our study also indicated that, compared to the control group, the study group had a better total effective rate, a lower recurrence rate, and a lower incidence of adverse reactions. This finding suggests that vitamin B12 injection combined with pulling, paravertebral positive spot cutaneous needle tapping, and cupping has a positive clinical effect in the treatment of lateral femoral cutaneous neuritis, as it can reduce the recurrence rate and the incidence of adverse reactions in patients. Similarly, Talaei pointed out that vitamin B12 was effective for the treatment of symptomatic painful diabetic neuropathy (40). However, it should be noted that we are not claiming that this combined therapy is superior to other treatments; rather, it shows promising results in this specific study context.

Our study has some limitations. First, it was a small single-center study. Due to the limited sample size and the single-center setting, the statistical power for detecting adverse events was relatively low. Second, our study did not incorporate a double-blind design. According to the Grading of Recommendations, Assessment, Development, and Evaluations (GRADE) system, the lack of blinding results in a moderate level of evidence quality. Since the patients were aware of the treatment they received, their subjective reports regarding the relief of pain and numbness might have been influenced by their expectations or psychological factors, potentially introducing evaluation bias. In addition, the follow-up period after treatment was relatively short. A short follow-up may not fully capture the long-term recurrence patterns of the disease. Some patients may experience a recurrence after a more extended period, which could not be accurately detected within the current follow-up timeframe. This limitation may lead to an overestimation of the long-term effectiveness of the treatment in preventing recurrence. To address these limitations, future research should conduct blinded multicenter and long-term trials.

## Conclusion

This study indicates that vitamin B12 injection, combined with pulling, paravertebral positive spot cutaneous needle tapping, and cupping, can significantly improve clinical outcomes with lateral femoral cutaneous neuritis. The combined therapy effectively alleviates pain and numbness, reduces recurrence rate, lowers the incidence of adverse reactions. However, larger-scale trials are needed to further confirm these findings.

## Data Availability

The original contributions presented in the study are included in the article/supplementary material, further inquiries can be directed to the corresponding author.
